# Extended Lattice Boltzmann Model

**DOI:** 10.3390/e23040475

**Published:** 2021-04-17

**Authors:** Mohammad Hossein Saadat, Benedikt Dorschner, Ilya Karlin

**Affiliations:** Department of Mechanical and Process Engineering, ETH Zurich, 8092 Zurich, Switzerland; msaadat@ethz.ch (M.H.S.); bdorschn@ethz.ch (B.D.)

**Keywords:** lattice Boltzmann method, Galilean invariance, extended equilibrium

## Abstract

Conventional lattice Boltzmann models for the simulation of fluid dynamics are restricted by an error in the stress tensor that is negligible only for small flow velocity and at a singular value of the temperature. To that end, we propose a unified formulation that restores Galilean invariance and the isotropy of the stress tensor by introducing an extended equilibrium. This modification extends lattice Boltzmann models to simulations with higher values of the flow velocity and can be used at temperatures that are higher than the lattice reference temperature, which enhances computational efficiency by decreasing the number of required time steps. Furthermore, the extended model also remains valid for stretched lattices, which are useful when flow gradients are predominant in one direction. The model is validated by simulations of two- and three-dimensional benchmark problems, including the double shear layer flow, the decay of homogeneous isotropic turbulence, the laminar boundary layer over a flat plate and the turbulent channel flow.

## 1. Introduction

The lattice Boltzmann method (LBM) solves a Boltzmann-type kinetic equation on a discrete velocity set, forming the links of a space-filling lattice. Efficiency of the LBM makes it attractive for the simulation of a wide range of problems in fluid dynamics, see, for example, [1,2].

In this paper, we revisit the restrictions of LBM due to the geometry of the discrete velocities. It is well known that standard LBM velocities yield a persistent error in the fluid stress tensor, which breaks Galilean invariance and limits the operation range of LBM to small flow velocities and a singular value of the lattice reference temperature; only under these conditions can the error be ignored. While one can cope with this error in most incompressible flow applications [1,2], it certainly affects high-speed compressible flows [3,4,5,6,7,8,9,10,11,12] and sometimes even low-speed isothermal cases [13]. Moreover, the same error is amplified when using stretched (rectangular) lattices instead of the conventional (cubic) lattice, where in addition to the corrupted Galilean invariance, the stress tensor becomes anisotropic [14,15].

The extension of LBM beyond its classical operation domain has so far been addressed with different techniques, depending on the desired outcome. Introducing lattices with more velocities (multi-speed lattices) [16] is one technique, which comes with a significant increase of computational cost and has been used mostly for high-speed compressible flow applications [16]. In the standard LB setting, on the other hand, one approach is to alter the relaxation rates and use a multi-relaxation time collision operator [9]. Another approach to extend the flow velocity and temperature range of the standard cubic lattices is to add correction terms to the original LBM [4,5,6,7,8,10,11,12,17,18,19,20,21]. The realization varies among different authors but none address the general case of rectangular lattices. On the other hand, rectangular lattices may improve the computational efficiency of the LBM by using a coarser mesh in the direction of smaller gradients in the flow. Unlike other approaches of handling non-uniform grids (e.g., Eulerian [22,23] and semi-Lagrangian off-lattice LBM [24,25,26,27] or grid refinement techniques [28,29]), stretched lattices do not require a substantial change in the standard LBM algorithm. Recent work on the stretched LBM restores the isotropy of the stress tensor by using multi-relaxation time LBM models [30,31,32]. However, these approaches do not address the flow velocity and temperature restrictions.

In this paper, we propose a unified view on the three aspects of the problem, the velocity range, the temperature range and grid stretching, which all stem from the same error, induced by constraints of the discrete velocity set. In particular, we propose to use an extended equilibrium, which restores the Galilean invariance and isotropy of the stress tensor, enabling simulations at higher flow velocities, higher temperatures using both cubic and stretched lattices, yielding increased accuracy and efficiency.

The paper is organized as follows—in Section 2, we start by presenting the discrete kinetic equations, following the standard single-relaxation time lattice Bhatnagar–Gross–Krook (LBGK) setting, as well as the equilibrium and extended equilibrium formulation, followed by the derivation of the model’s hydrodynamic limit. In addition, the locally corrected LBM of Reference [9] is compared to the extended LBGK in Appendix A.

Subsequently, in Section 3, we assess the validity, accuracy and performance of our model using both two- and three-dimensional benchmark problems. As a first step, we verify Galilean invariance, temperature independence and isotropy of the model on the example of an advected decaying shear wave. It is shown that the theoretical viscosity is recovered for both cubic as well as stretched lattices in a large range of temperatures and advection velocities. This also indicates that the model can readily be extended to high-speed compressible flows, provided that it is augmented with a suitable solver for the total energy. Next, for the example of homogeneous isotropic turbulence, we demonstrate that a speed-up can be achieved by using an operating temperature, which is larger than the lattice reference temperature. The present model can also be viewed as an alternative to preconditioned LBM [33] for accelerating the convergence rate but without the restriction to steady flows. Finally, accuracy and performance are assessed for rectangular lattices using the doubly periodic shear flow, laminar flow over a flat plate and turbulent channel flow as examples. Conclusions are drawn in Section 4.

## 2. Discrete Kinetic Equations

### 2.1. LBGK

We consider the LBGK equation for the populations $f_{i}$, associated to the discrete velocities $v_{i}$ for i=0,…,Q−1,

(1) $$\begin{array}{c} f_{i}\left(x+v_{i}\delta t,t+\delta t\right)-f_{i}\left(x,t\right)=\omega \left(f_{i}^{\mathrm{ex}}-f_{i}\right), \end{array}$$

where x denotes the location in space and δt is the time step. The extended equilibrium $f_{i}^{\mathrm{ex}}$, which will be specified below, satisfies the local conservation laws for the density ρ and momentum ρu,

(2) $$\begin{array}{cc} \rho & =\sum_{i=0}^{Q-1}f_{i}^{\mathrm{ex}}=\sum_{i=0}^{Q-1}f_{i}, \end{array}$$

(3) $$\begin{array}{cc} \rho u & =\sum_{i=0}^{Q-1}v_{i}f_{i}^{\mathrm{ex}}=\sum_{i=0}^{Q-1}v_{i}f_{i}. \end{array}$$

As we will show below, the relaxation parameter ω is related to the kinematic viscosity ν,

(4) $$\begin{array}{c} \nu =\left(\frac{1}{\omega }-\frac{1}{2}\right)RT\delta t, \end{array}$$

where *T* is the temperature and *R* is the gas constant. We now proceed with identifying the extended equilibrium.

### 2.2. Discrete Velocities and Factorization

We use the D3Q27 lattice, where D=3 denotes the spatial dimension and Q=27 is the number of discrete speeds, which are given by

(5) $$c_{i}=\left(c_{ix},c_{iy},c_{iz}\right),\;c_{i\alpha }\in \left\{-1,0,1\right\}.$$

With (5), we define the particles’ velocities $v_{i}$ in a stretched cell as

(6) $$v_{i}=\left(\lambda_{x}c_{ix},\lambda_{y}c_{iy},\lambda_{z}c_{iz}\right),$$

where $\lambda_{\alpha }$ is the stretching factor in the direction α. To maintain on-lattice propagation, the cell size is changed accordingly to $\delta x_{\alpha }=\lambda_{\alpha }\delta t$.

The (normalized, $M_{000}=1$) moments $M_{lmn}$ are defined using the convention

(7) l→x,m→y,n→z;l,m,n=0,1,2,…,

and thus

(8) $$\rho M_{lmn}=\sum_{i=0}^{Q-1}v_{ix}^{l}v_{iy}^{m}v_{iz}^{n}f_{i}.$$

For convenience, we use a more specific notation for the first-order and the diagonal second-order moments,

(9) $$\begin{array}{cc} \; & M_{100}=u_{x},\;M_{010}=u_{y},\;M_{001}=u_{z}, \end{array}$$

(10) $$\begin{array}{cc} \; & M_{200}=P_{xx},\;M_{020}=P_{yy},\;M_{002}=P_{zz}. \end{array}$$

We essentially follow [34] and consider a class of factorized populations. To that end, we define a triplet of functions in the three variables, $u_{\alpha }$, $P_{\alpha \alpha }$ and $\lambda_{\alpha }$,

(11) $$\begin{array}{cc} \Psi_{0}\left(u_{\alpha },P_{\alpha \alpha },\lambda_{\alpha }\right) & =1-\frac{P_{\alpha \alpha }}{\lambda_{\alpha }^{2}}, \end{array}$$

(12) $$\begin{array}{cc} \Psi_{1}\left(u_{\alpha },P_{\alpha \alpha },\lambda_{\alpha }\right) & =\frac{1}{2}\left(\frac{u_{\alpha }}{\lambda_{\alpha }}+\frac{P_{\alpha \alpha }}{\lambda_{\alpha }^{2}}\right), \end{array}$$

(13) $$\begin{array}{cc} \Psi_{-1}\left(u_{\alpha },P_{\alpha \alpha },\lambda_{\alpha }\right) & =\frac{1}{2}\left(-\frac{u_{\alpha }}{\lambda_{\alpha }}+\frac{P_{\alpha \alpha }}{\lambda_{\alpha }^{2}}\right). \end{array}$$

For the vectors u, 𝓟, and λ,

(14) $$\begin{array}{cc} u & =(u_{x},u_{y},u_{z}), \end{array}$$

(15) $$\begin{array}{cc} 𝓟 & =(P_{xx},P_{yy},P_{zz}), \end{array}$$

(16) $$\begin{array}{cc} \lambda & =(\lambda_{x},\lambda_{y},\lambda_{z}), \end{array}$$

we consider a product-form, associated with the discrete velocities $v_{i}$ (6),

(17) $$\Psi_{i}\left(u,𝓟,\lambda \right)=\prod_{\alpha =x,y,z}\Psi_{c_{i\alpha }}\left(u_{\alpha },P_{\alpha \alpha },\lambda_{\alpha }\right).$$

The normalized moments of the product-form (17),

(18) $$M_{lmn}=\sum_{i=0}^{Q-1}v_{ix}^{l}v_{iy}^{m}v_{iz}^{n}\Psi_{i},$$

are readily computed thanks to the factorization,

(19) $$M_{lmn}=M_{l00}M_{0m0}M_{00n},$$

where

(20) $$\begin{array}{cc} M_{000} & =1, \end{array}$$

(21) $$\begin{array}{cc} M_{l00} & =\left\{\begin{array}{cc} \lambda_{x}^{l-1}u_{x},\; & l\;\mathrm{odd}\\ \lambda_{x}^{l-2}P_{xx},\; & l\;\mathrm{even} \end{array}\right., \end{array}$$

(22) $$\begin{array}{cc} M_{0m0} & =\left\{\begin{array}{cc} \lambda_{y}^{m-1}u_{y},\; & m\;\mathrm{odd}\\ \lambda_{y}^{m-2}P_{yy},\; & m\;\mathrm{even} \end{array}\right., \end{array}$$

(23) $$\begin{array}{cc} M_{00n} & =\left\{\begin{array}{cc} \lambda_{z}^{n-1}u_{z},\; & n\;\mathrm{odd}\\ \lambda_{z}^{n-2}P_{zz},\; & n\;\mathrm{even} \end{array}\right.. \end{array}$$

For any stretching (16), the six-parametric family of normalized populations (17) is identified by the flow velocity (14) and the diagonal of the pressure tensor at unit density (15), and was termed the unidirectional quasi-equilibrium in Ref. [34]. We make use of the product-form (17) to construct all pertinent populations, the equilibrium and the extended equilibrium.

### 2.3. Equilibrium and Extended Equilibrium

The equilibrium $f_{i}^{\mathrm{eq}}$ is defined by setting $P_{\alpha \alpha }$ (10) equal to the equilibrium diagonal element of the pressure tensor at unit density,

(24) $$\begin{array}{c} P_{\alpha \alpha }^{\mathrm{eq}}=RT+u_{\alpha }^{2}. \end{array}$$

Substituting (24) into (17), we get

(25) $$\begin{array}{cc} f_{i}^{\mathrm{eq}} & =\rho \Psi_{i}\left(u,{𝓟}^{\mathrm{eq}},\lambda \right). \end{array}$$

With (18), we find the pressure tensor and the third-order moment tensor at the equilibrium (25) as follows,

(26) $$\begin{array}{cc} P^{\mathrm{eq}} & =\sum_{i=0}^{Q-1}v_{i}⊗v_{i}f_{i}^{\mathrm{eq}}=P^{\mathrm{MB}}, \end{array}$$

(27) $$\begin{array}{cc} Q^{\mathrm{eq}} & =\sum_{i=0}^{Q-1}v_{i}⊗v_{i}⊗v_{i}f_{i}^{\mathrm{eq}}=Q^{\mathrm{MB}}+\tilde{Q}. \end{array}$$

The isotropic parts, $P^{\mathrm{MB}}$ and $Q^{\mathrm{MB}}$, are the Maxwell–Boltzmann (MB) pressure tensor and the third-order moment tensor, respectively,

(28) $$\begin{array}{cc} P^{\mathrm{MB}} & =pI+\rho u⊗u, \end{array}$$

(29) $$\begin{array}{cc} Q^{\mathrm{MB}} & =\mathrm{sym}(pI⊗u)+\rho u⊗u⊗u, \end{array}$$

where p=ρRT is the pressure, sym(…) denotes symmetrization and I is the unit tensor. The anisotropy of the equilibrium (25) manifests with the deviation $\tilde{Q}=Q^{\mathrm{eq}}-Q^{\mathrm{MB}}$ in (29), where only the diagonal elements are non-vanishing,

(30) $$\begin{array}{c} {\tilde{Q}}_{\alpha \beta \gamma }=\left\{\begin{array}{ccc} \rho u_{\alpha }\left(\lambda_{\alpha }^{2}-3RT\right)-\rho u_{\alpha }^{3}, & \;\mathrm{if}\;\alpha =\beta =\gamma , & \\ 0, & \mathrm{otherwise}. & \end{array}\right. \end{array}$$

The origin of the *diagonal anomaly* (30) is the geometric constraint, $v_{i\alpha }^{3}=\lambda_{\alpha }^{2}v_{i\alpha }$, which is imposed by the choice of the discrete speeds (5), and is well known in the case of the standard (unstretched) lattice with $\lambda_{\alpha }=1$. A remedy in the latter case is to minimize spurious effects of anisotropy by fixing the temperature $T=T_{L}$,

(31) $$RT_{L}=\frac{1}{3},$$

In order to eliminate the linear term $∼u_{\alpha }$ in (30). Thus, using the equilibrium (25) in the LBGK Equation (1) imposes a two-fold restriction on the operation domain: the temperature cannot be chosen differently from (31) and the flow velocity has to be maintained asymptotically vanishing. Moreover, for stretched lattices, the anisotropy becomes even more pronounced since it is not possible to eliminate the linear deviation in all directions simultaneously by fixing any temperature.

Alternatively, the spurious anisotropy effects can be canceled out by extending the equilibrium such that the third-order moment anomaly is compensated in the hydrodynamic limit. Because the anomaly only concerns the diagonal (unidirectional) elements of the third-order moments, the cancellation can be achieved by redefining the diagonal elements of the second-order moments. As demonstrated below, in order to cancel the errors, the diagonal elements $P_{\alpha \alpha }^{\mathrm{ex}}$ for the extended equilibrium must be chosen as

(32) $$\begin{array}{c} P_{\alpha \alpha }^{\mathrm{ex}}=P_{\alpha \alpha }^{\mathrm{eq}}+\delta t\left(\frac{2-\omega }{2\rho \omega }\right)\partial_{\alpha }{\tilde{Q}}_{\alpha \alpha \alpha }, \end{array}$$

where spatial derivative is evaluated using a second-order central difference scheme. Hence, the extended equilibrium $f_{i}^{\mathrm{ex}}$ is specified by using the product-form (17),

(33) $$\begin{array}{cc} f_{i}^{\mathrm{ex}} & =\rho \Psi_{i}\left(u,{𝓟}^{\mathrm{ex}},\lambda \right). \end{array}$$

We shall now proceed with the derivation of the Navier–Stokes equations in the hydrodynamic limit of the proposed extended LBGK model.

### 2.4. Hydrodynamic Limit with Extended Equilibrium

Taylor expansion of the shift operator in (1) to second order gives,

(34) $$\left[\delta tD_{i}+\frac{\delta t^{2}}{2}D_{i}D_{i}\right]f_{i}=\omega \left(f_{i}^{\mathrm{ex}}-f_{i}\right),$$

where $D_{i}$ is the derivative along the characteristics,

(35) $$D_{i}=\partial_{t}+v_{i}\cdot \nabla .$$

Introducing the multi-scale expansion,

(36) $$\begin{array}{cc} f_{i} & =f_{i}^{\left(0\right)}+\delta tf_{i}^{\left(1\right)}+\delta t^{2}f_{i}^{\left(2\right)}+O\left(\delta t^{3}\right), \end{array}$$

(37) $$\begin{array}{cc} f_{i}^{\mathrm{ex}} & =f_{i}^{\mathrm{ex}(0)}+\delta tf_{i}^{\mathrm{ex}(1)}+\delta t^{2}f_{i}^{\mathrm{ex}(2)}+O\left(\delta t^{3}\right), \end{array}$$

(38) $$\begin{array}{cc} \partial_{t} & =\partial_{t}^{\left(1\right)}+\delta t\partial_{t}^{\left(2\right)}+O\left(\delta t^{2}\right), \end{array}$$

substituting into (34) and using the notation,

(39) $$\begin{array}{cc} \; & D_{i}^{\left(1\right)}=\partial_{t}^{\left(1\right)}+v_{i}\cdot \nabla , \end{array}$$

we obtain, from the zeroth to second order in the time step δt,

(40) $$\begin{array}{cc} \; & f_{i}^{\left(0\right)}=f_{i}^{\mathrm{ex}(0)}=f_{i}^{\mathrm{eq}}, \end{array}$$

(41) $$\begin{array}{cc} \; & D_{i}^{\left(1\right)}f_{i}^{\left(0\right)}=-\omega f_{i}^{\left(1\right)}+\omega f_{i}^{\mathrm{ex}(1)}, \end{array}$$

(42) $$\begin{array}{cc} \; & \partial_{t}^{\left(2\right)}f_{i}^{\left(0\right)}+v_{i}\cdot \nabla f_{i}^{\left(1\right)}-\frac{\omega }{2}D_{i}^{\left(1\right)}f_{i}^{\left(1\right)}+\frac{\omega }{2}D_{i}^{\left(1\right)}f_{i}^{\mathrm{ex}(1)}=-\omega f_{i}^{\left(2\right)}+\omega f_{i}^{\mathrm{ex}(2)}. \end{array}$$

With (40), the mass and the momentum conservation (2) and (3) imply the solvability conditions,

(43) $$\begin{array}{cc} \; & \sum_{i=0}^{Q-1}f_{i}^{\mathrm{ex}(k)}=\sum_{i=0}^{Q-1}f_{i}^{\left(k\right)}=0,\;k=1,2\ldots ; \end{array}$$

(44) $$\begin{array}{cc} \; & \sum_{i=0}^{Q-1}v_{i}f_{i}^{\mathrm{ex}(k)}=\sum_{i=0}^{Q-1}v_{i}f_{i}^{\left(k\right)}=0,\;k=1,2,\ldots . \end{array}$$

With the equilibrium (25), taking into account the solvability conditions (43) and (44), and also making use of the equilibrium pressure tensor (26) and (28), the first-order Equation (41) implies the following relations for the density and the momentum,

(45) $$\begin{array}{cc} \; & \partial_{t}^{\left(1\right)}\rho =-\nabla \cdot \left(\rho u\right), \end{array}$$

(46) $$\begin{array}{cc} \; & \partial_{t}^{\left(1\right)}\left(\rho u\right)=-\nabla \cdot \left(pI+\rho u⊗u\right). \end{array}$$

Moreover, the first-order constitutive relation for the nonequilibrium pressure tensor $P^{\left(1\right)}$ is found from (41) as follows,

(47) $$\begin{array}{cc} \; & -\omega P^{\left(1\right)}+\omega P^{\mathrm{ex}(1)}=\partial_{t}^{\left(1\right)}P^{\mathrm{eq}}+\nabla \cdot Q^{\mathrm{eq}}, \end{array}$$

where

(48) $$\begin{array}{cc} \; & P^{\left(1\right)}=\sum_{i=0}^{Q-1}v_{i}⊗v_{i}f_{i}^{\left(1\right)}, \end{array}$$

(49) $$\begin{array}{cc} \; & P^{\mathrm{ex}(1)}=\sum_{i=0}^{Q-1}v_{i}⊗v_{i}f_{i}^{\mathrm{ex}(1)}. \end{array}$$

With the help of Equations (28) and (29), the first-order constitutive relation (47) is transformed to make explicit the contribution of the anomalous term (30),

(50) $$\begin{array}{c} -\omega P^{\left(1\right)}+\omega P^{\mathrm{ex}(1)}=\nabla \cdot \tilde{Q}+\left(\partial_{t}^{\left(1\right)}P^{\mathrm{MB}}+\nabla \cdot Q^{\mathrm{MB}}\right). \end{array}$$

The last term is evaluated using (45) and (46) to give,

(51) $$\partial_{t}^{\left(1\right)}P^{\mathrm{MB}}+\nabla \cdot Q^{\mathrm{MB}}=\rho RT\left(\nabla u+\nabla u^{†}\right),$$

where ${\left(\cdot \right)}^{†}$ denotes transposition. Combining (51) and (50), the first-order constitutive relation becomes,

(52) $$\begin{array}{c} -\omega P^{\left(1\right)}=\left(\nabla \cdot \tilde{Q}-\omega P^{\mathrm{ex}(1)}\right)+\rho RT\left(\nabla u+\nabla u^{†}\right). \end{array}$$

Note that, if we had used the equilibrium $f_{i}^{\mathrm{eq}}$ instead of the extended equilibrium $f_{i}^{\mathrm{ex}}$ in (1), at this stage of the derivation, we get instead of (52),

(53) $$\begin{array}{c} -\omega P^{\left(1\right)}=\nabla \cdot \tilde{Q}+\rho RT\left(\nabla u+\nabla u^{†}\right). \end{array}$$

The anomalous term $\nabla \cdot \tilde{Q}$ cannot be canceled in the latter expression, rather, by choosing $T=T_{L}$ (31), its effect can be ignored but only under the assumption of an asymptotically small flow velocity. Moreover, for a quasi-incompressible flow (Ma→0, density variation $\nabla \rho ∼{\mathrm{Ma}}^{2}$, where Ma is a characteristic Mach number), it is possible to further reduce the effect of the anomaly by rescaling the relaxation parameter [9], see a discussion in Appendix A.

In a contrast, using the present formulation, the cancellation is possible by finding the corresponding expression for the correction term $P^{\mathrm{ex}(1)}$, to which end we need to consider the second-order contribution to the momentum equation. Applying the solvability condition (43) and (44) to the second-order Equation (42), we obtain,

(54) $$\begin{array}{cc} \; & \partial_{t}^{\left(2\right)}\rho =0, \end{array}$$

(55) $$\begin{array}{cc} \; & \partial_{t}^{\left(2\right)}\left(\rho u\right)=-\nabla \cdot \left[\left(1-\frac{\omega }{2}\right)P^{\left(1\right)}+\frac{\omega }{2}P^{\mathrm{ex}(1)}\right]. \end{array}$$

The latter is transformed by virtue of (52),

(56) $$\begin{array}{cc} \partial_{t}^{\left(2\right)}\left(\rho u\right)= & -\nabla \cdot \left[-\left(\frac{1}{\omega }-\frac{1}{2}\right)\rho RT\left(\nabla u+\nabla u^{†}\right)\right]+\nabla \cdot \left[\left(\frac{1}{\omega }-\frac{1}{2}\right)\nabla \cdot \tilde{Q}-P^{\mathrm{ex}(1)}\right]. \end{array}$$

The last (anomalous) term is canceled out by choosing,

(57) $$\begin{array}{c} P^{\mathrm{ex}(1)}=\left(\frac{2-\omega }{2\omega }\right)\nabla \cdot \tilde{Q}. \end{array}$$

Combining the result (57) with the zeroth-order (equilibrium) value, we arrive at the extended pressure tensor

(58) $$\begin{array}{cc} P^{\mathrm{ex}} & =P^{\mathrm{eq}}+\delta tP^{\mathrm{ex}(1)}\\ \; & =pI+\rho u⊗u+\delta t\left(\frac{2-\omega }{2\omega }\right)\nabla \cdot \tilde{Q}. \end{array}$$

Since the anomalous contribution is a diagonal tensor, cf. Equation (30), the result (58) is implemented with the extended equilibrium in the product-form by choosing the normalized (at unit density) diagonal elements of the pressure tensor as follows,

(59) $$\begin{array}{cc} P_{\alpha \alpha }^{\mathrm{ex}} & =RT+u_{\alpha }^{2}+\delta t\left(\frac{2-\omega }{2\rho \omega }\right)\partial_{\alpha }\left(\rho u_{\alpha }\left(\lambda_{\alpha }^{2}-3RT-u_{\alpha }^{2}\right)\right), \end{array}$$

which is equivalent to (32). Finally, combining the first- and second-order contributions to the density and the momentum equation, (45), (46), (54) and (56), using a notation, $\partial_{t}=\partial_{t}^{\left(1\right)}+\delta t\partial_{t}^{\left(2\right)}$, and also taking into account the cancellation of the anomalous term in (56), we arrive at the continuity and the flow equations as follows,

(60) $$\begin{array}{cc} \; & \partial_{t}\rho +\nabla \cdot \left(\rho u\right)=0, \end{array}$$

(61) $$\begin{array}{cc} \; & \partial_{t}u+u\cdot \nabla u+\frac{1}{\rho }\nabla p+\frac{1}{\rho }\nabla \cdot \Pi =0, \end{array}$$

where *p* is the pressure of ideal gas at constant temperature *T*,

(62) $$\begin{array}{c} p=\rho RT, \end{array}$$

Π is the viscous pressure tensor,

(63) $$\begin{array}{c} \Pi =-\mu S, \end{array}$$

with S the rate of strain,

(64) $$\begin{array}{c} S=\nabla u+\nabla u^{†}, \end{array}$$

and μ the dynamic viscosity,

(65) $$\begin{array}{c} \mu =\left(\frac{1}{\omega }-\frac{1}{2}\right)p\delta t. \end{array}$$

The above considerations can be summarized as follows—because of the third-order moment anomaly (30), the LBGK Equation (1) with the product-form equilibrium (25) is restricted in several ways, namely:

(i) The temperature is restricted to a single value, the lattice reference temperature $T_{L}$ (31);
(ii) The flow velocity has to be asymptotically vanishing;
(iii) Stretched velocities amplify these restrictions by making it impossible to cancel even the linear (in velocity) anomaly in all the directions simultaneously.

Note that, in addition to all of the restrictions above, when using the conventional second-order equilibrium obtained by retaining the terms up to the order of $∼u_{\alpha }u_{\beta }$ in (25), the anomaly becomes not only confined to the diagonal elements $Q_{\alpha \alpha \alpha }^{\mathrm{eq}}$ but also contaminates the off-diagonal elements $Q_{\alpha \beta \beta }^{\mathrm{eq}}$. While the diagonal anomaly (30) is genuine, that is, it is caused by the geometry of the discrete velocities, this additional off-diagonal deviation is due to an unsolicited second-order truncation of the product-form equilibrium (25).

The proposed revision of the LBGK model is based on extending the product-form equilibrium such that the anomaly of the diagonal third-order moment is compensated in the hydrodynamic limit by counter terms, which are added to the diagonal of the equilibrium pressure tensor. With this, all three restrictions mentioned above are addressed at once, without making a special distinction between the temperature, flow velocity or stretching as separate causes for the anisotropy.

## 3. Numerical Results

In this section, we shall access the accuracy and performance of the proposed LB model in a variety of scenarios of activating spurious anisotropy. First, we test Galilean invariance, isotropy and temperature independence of the model with both regular and rectangular lattices in the simulation of a decaying shear wave. Second, we validate the model for the more complex case of decaying homogeneous isotropic turbulence and show the effectiveness of using higher temperatures in saving compute time. Third, we investigate the applicability of the proposed model with stretched lattices in a periodic double shear layer flow, in a laminar flow over a flat plate, and finally in the case of the turbulent channel flow. In the simulations below, the gas constant was set to R=1, the time step is δt=1 and Grad’s approximation, as proposed in [35], was used for wall boundary conditions.

### 3.1. Galilean Invariance, Isotropy and Temperature Independence Test

To probe the Galilean invariance and temperature independence of the model, the kinematic viscosity ν=μ/ρ (4) is measured for the decay of a plane shear wave. First, we consider the axis-aligned setup, with the initial condition,

(66) $$\rho =\rho_{0},\;u_{x}=a_{0}\sin\left(2\pi y/L_{y}\right),\;u_{y}=\mathrm{Ma}\sqrt{T},$$

where $\mathrm{Ma}=u_{0}/\sqrt{T}$ is the advection Mach number, $a_{0}=0.001$ is the amplitude, $L_{y}=200$ is number of grid nodes in the *y* direction, $\rho_{0}=1$. The nominal kinematic viscosity is set to ν=0.01. Periodic boundary conditions are imposed in both *x*- and *y*- directions. The numerical viscosity ($\nu_{num}$) is measured by fitting an exponential to the time decay of maximum flow velocity $u_{x,\max}∼e^{-\nu t{\left(2\pi /L_{y}\right)}^{2}}$. In this special case, the diagonal anomaly (30) is dormant and does not trigger any spurious effects because the derivatives $\partial_{x}{\tilde{Q}}_{xxx}$ and $\partial_{y}{\tilde{Q}}_{yyy}$ both vanish. Consequently, the extended equilibrium (33) becomes equivalent to the product-form equilibrium (25) in this case.

**Figure 1:**
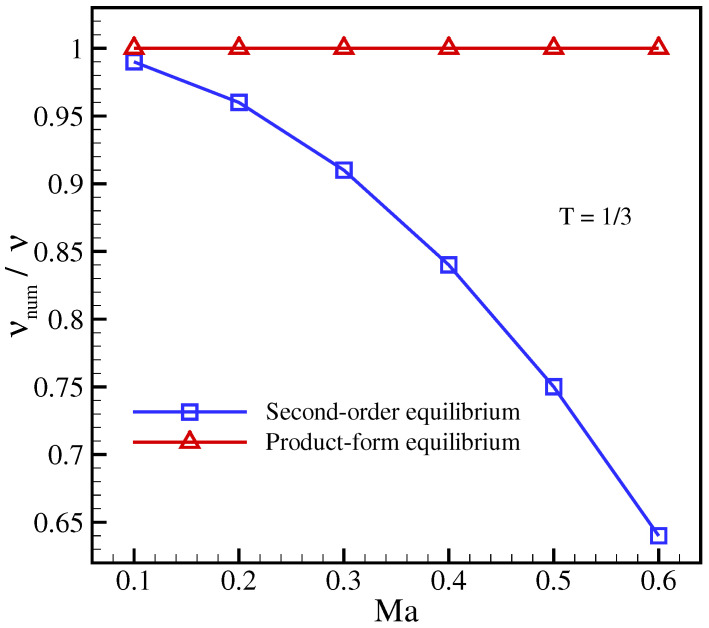
Numerical measurement of viscosity for axis-aligned setup at temperature T=1/3 for different velocities. The exact solution corresponds to $\nu_{num}/\nu =1$.

**Figure 2:**
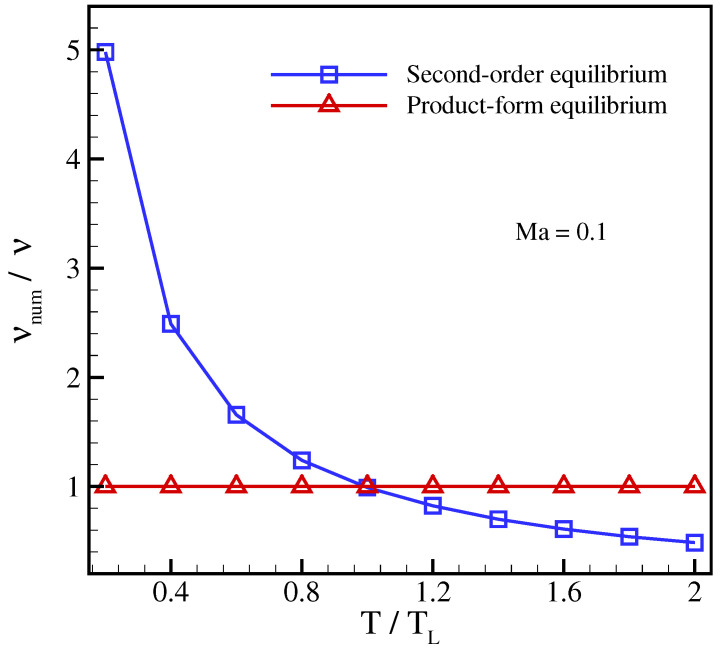
Numerical measurement of viscosity for axis-aligned setup at Mach number Ma=0.1 for different temperatures. The exact solution corresponds to $\nu_{num}/\nu =1$.

In order to compare with the standard LBGK, the standard velocities $\lambda_{\alpha }=1$ were used in this simulation. Figure 1 and Figure 2 show the importance of using the product-form equilibrium (25) as opposed to the conventional LBGK model with the second-order equilibrium. A strong dependence of the viscosity on the reference frame for the second-order equilibrium can be seen in Figure 1, where the viscosity drops with increasing advection Mach number. This well-known artifact of the second-order equilibrium is due to the non-vanishing anomaly in the off-diagonal moments $Q_{\alpha \beta \beta }^{\mathrm{eq}}$ and, unlike the diagonal anomaly, is caused only by the approximate treatment of the product-form equilibrium. Moreover, as shown in Figure 2, even at a small enough velocity this spurious feature improves only at the lattice reference temperature $T_{L}$. In contrast, as is shown in Figure 1 and Figure 2, the product-form equilibrium of the present model is able to accurately predict the viscosity in this setup for a wide range of temperatures and reference frame velocities.

**Figure 3:**
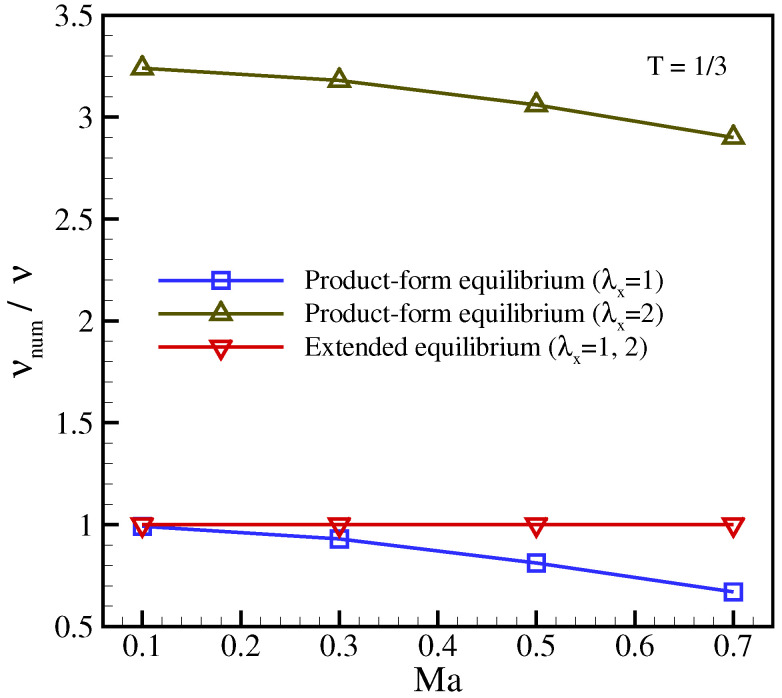
Numerical measurement of viscosity for rotated setup at temperature T=1/3 for different velocities and stretching ratios. The exact solution corresponds to $\nu_{num}/\nu =1$.

Next, in order to trigger the anisotropy of the deviation terms (30) and to show the necessity of using the extended equilibrium, the shear wave is rotated by π/4. The anisotropy is further increased by also conducting simulations on a stretched grid with $\lambda_{x}=2$. The temperature is kept at T=1/3. The viscosity measurement is shown in Figure 3 for different advection Mach numbers and stretching factors. It can be observed that the model lacks Galilean invariance for larger velocities when using the product-form equilibrium without correction (25). Furthermore, the stretching factor $\lambda_{x}=2$ results in a significant hyper-viscosity since the deviation (30) in this case amounts to a large positive number. However, once the correction term is included and the extended equilibrium (33) is used, the present model recovers the imposed viscosity, independent of the frame velocity and stretching factor.

### 3.2. Decaying Homogeneous Isotropic Turbulence

In order to further validate the model as a reliable method for the simulation of complex flows and to show the application of using higher temperatures, decaying homogeneous isotropic turbulence was considered. The initial condition, in a box of the size L×L×L, was set at unit density and constant temperature along with a divergence-free velocity field, which follows the specified energy spectrum,

(67) $$\begin{array}{cc} E(\kappa ) & =A\kappa^{4}e^{-2{\left(\kappa /\kappa_{0}\right)}^{2}}, \end{array}$$

where κ is the wave number, $\kappa_{0}$ is the wave number at which the spectrum peaks and *A* is the parameter that controls the initial kinetic energy [36]. The initial velocity field is generated using a kinematic simulation as proposed in [37]. The turbulent Mach number is defined as

(68) $$\begin{array}{c} {\mathrm{Ma}}_{t}=\frac{\sqrt{\;\bar{\;u\cdot u\;}\;}}{c_{s}}, \end{array}$$

where $c_{s}=\sqrt{T}$ is the speed of sound. The Reynolds number is based on the Taylor microscale,

(69) $$\begin{array}{c} \Lambda^{2}=\frac{u_{\mathrm{rms}}^{2}}{\;\bar{\;{\left(\partial_{x}u_{x}\right)}^{2}\;}\;}, \end{array}$$

and is given by

(70) ReΛ=ρ¯urmsΛμ,

where $u_{\mathrm{rms}}=\sqrt{\;\bar{\;u\cdot u\;}\;/3}$ is the root mean square (rms) of the velocity and overbar denotes the volume average over the entire computational domain.

**Figure 4:**
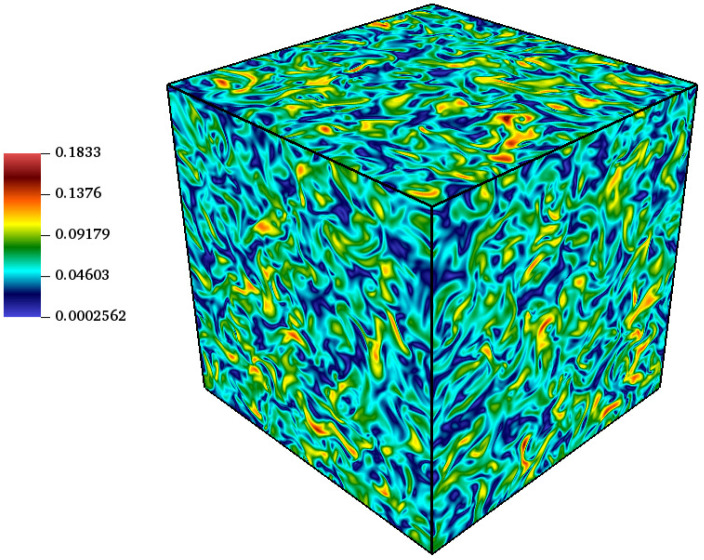
Velocity magnitude in lattice units for the decaying homogeneous isotropic turbulence at ${\mathrm{Ma}}_{t}=0.1$, ReΛ=72 and $t^{*}=1.0$ with temperature T=0.55.

Simulations were performed at ${\mathrm{Ma}}_{t}=0.1$, ReΛ=72, $\kappa_{0}=16\pi /L$, at two different temperatures, T=1/3 and T=0.55, and with L=256 grid points. Figure 4 shows a snapshot of the velocity magnitude $\sqrt{u\cdot u}$ at time $t^{*}=t/\tau =1.0$, where $\tau =L_{I}/u_{\mathrm{rms},0}$ is the eddy turnover time, which is defined based on the initial rms of the velocity and the integral length scale $L_{I}=\sqrt{2\pi }/\kappa_{0}$.

**Figure 5:**
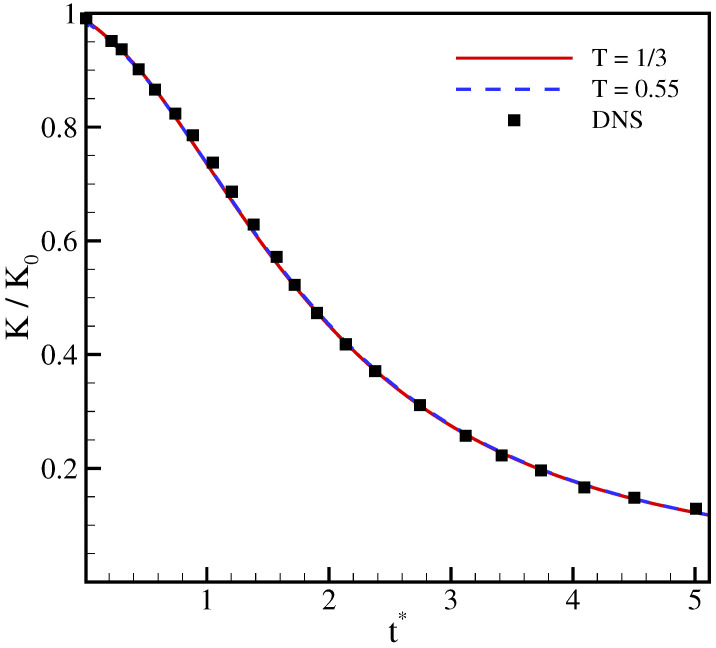
Time evolution of the turbulent kinetic energy for decaying isotropic turbulence at ${\mathrm{Ma}}_{t}=0.1$, ReΛ=72. Lines: present model; symbol: DNS [36].

**Figure 6:**
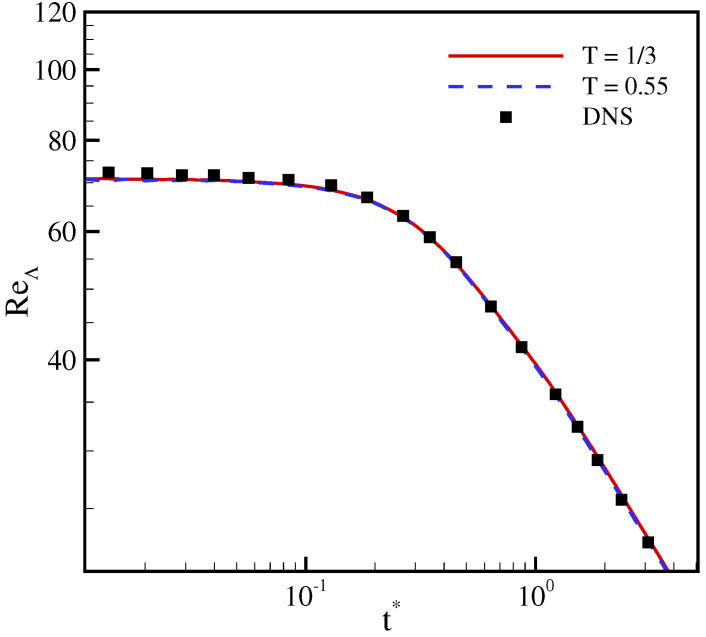
Time evolution of the Taylor microscale Reynolds number for decaying isotropic turbulence at ${\mathrm{Ma}}_{t}=0.1$, ReΛ=72. Lines: present model; symbol: direct numerical simulations (DNS) [36].

To quantitatively assess the accuracy of the model at different temperatures, the time evolution of the turbulent kinetic energy,

(71) $$\begin{array}{c} K=\frac{3}{2}u_{\mathrm{rms}}^{2}, \end{array}$$

normalized with its initial value ($K_{0}$), and of the Taylor microscale Reynolds number are compared in Figure 5 and Figure 6 with results from direct numerical simulations (DNS) [36]. It is apparent that the two working temperatures yields almost identical results that agree well with the DNS simulation. This indicates that the correction terms do not degrade the accuracy of the model at higher temperatures, even though the magnitude of error term (30) is higher due to amplification of the linear term.

The immediate advantage of using the present model at a temperature higher than the lattice temperature $T_{L}=1/3$ is that it effectively increases the characteristic velocity (here $u_{rms,0}$) and therefore the time step by a factor of $\sqrt{T/T_{L}}$. A larger time step is equivalent to fewer number of time steps. The present model, therefore, speeds up the simulation by a factor of $\sqrt{T/T_{L}}$ compared to the conventional LBM, which can operate only at the lattice temperature $T_{L}$. Furthermore, this speedup strategy can be used for both steady and unsteady flows. This is in contrast to the preconditioned LBM [33], which works by altering the effective Mach number and therefore reduces the disparity between the speeds of the acoustic wave propagation and the waves propagating with the fluid velocity, cf. [33]. This makes preconditioned LBM restricted to steady state applications. In contrast, the present model enables us to increase the speed of sound without changing the Mach number. This increases the effective time step of the solver. Therefore, the present model increases the computational efficiency by decreasing the number of required time steps. Note that the theoretical temperature range of the model (like any other models based on the D1Q3 lattice) is 0≤T≤1, beyond that the populations become negative and the model is unstable. Therefore, while small temperature is possible but not beneficial, large temperature greater than 1 is out of the stability domain

### 3.3. Periodic Double Shear Layer

The next validation case to test the accuracy of the proposed model with the stretched lattice is the periodic double shear layer flow with the initial condition,

(72) $$\begin{array}{cc} u_{x} & =\left\{\begin{array}{cc} u_{0}\tanh\left(\alpha \left(y/L-0.25\right)\right), & y\leq L/2,\\ u_{0}\tanh\left(\alpha \left(0.75-y/L\right)\right), & y>L/2, \end{array}\right. \end{array}$$

(73) $$\begin{array}{cc} u_{y} & =\delta u_{0}\sin\left(2\pi \left(x/L+0.25\right)\right), \end{array}$$

where *L* is the domain length in both *x* and *y* directions, $u_{0}=0.1$ is characteristic velocity, δ=0.05 is a perturbation of the *y*-velocity and α=80 controls the width of the shear layer. The Reynolds number is set to Re=u0L/ν=104 and the temperature is T=1/3.

**Figure 7:**
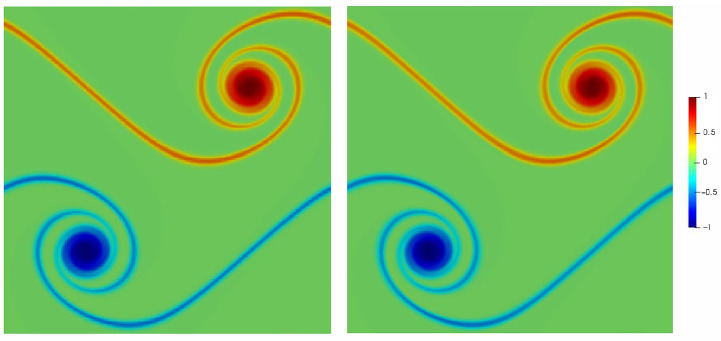
Vorticity field for double shear layer flow at $t^{*}=1$ with regular lattice (**left**) and stretched lattice (**right**). Vorticity magnitude is normalized by its maximum value.

Figure 7 shows the vorticity field at non-dimensional time $t^{*}=tu_{0}/L=1$ using the conventional square lattice $\lambda_{x}=\lambda_{y}=1$ and the rectangular lattice with $\lambda_{x}=2$, $\lambda_{y}=1$. Both lattice models perform qualitatively the same.

**Figure 8:**
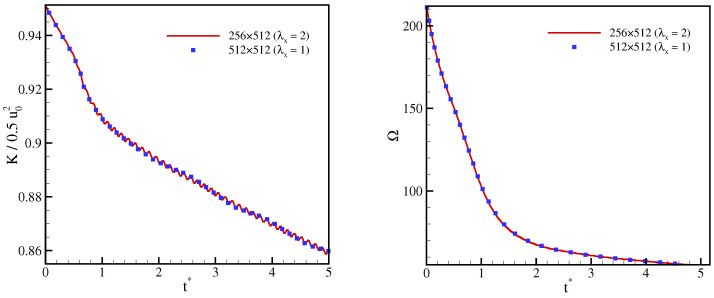
Evolution of kinetic energy (**left**) and enstrophy (**right**) for double shear layer flow at $Re=10^{4}$.

To quantify the effect of stretching on the accuracy, the time evolution of the mean kinetic energy and of the mean enstrophy $\Omega =\;\bar{\;\omega^{2}\;}\;/\frac{u_{0}^{2}}{L^{2}}$, with ω the vorticity magnitude, are compared in Figure 8. The results show only minor discrepancies, which indicates the validity of the model also on stretched meshes.

### 3.4. Laminar Boundary Layer over a Flat Plate

**Figure 9:**
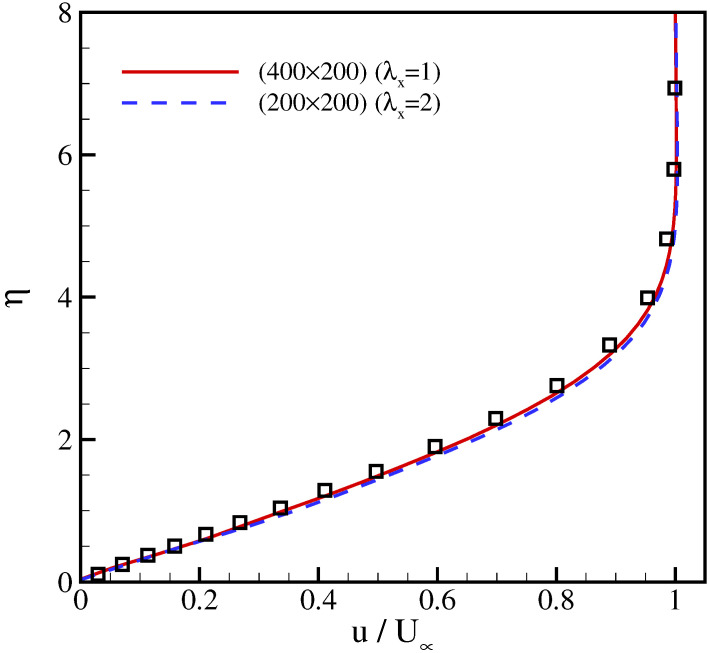
Comparison of the velocity profile at $x=L_{x}$ for flow over a flat plate at different stretching ratios. Lines: present model; symbols: Blasius solution.

The next test case validates our model for wall-bounded flows. We consider the laminar flow over a flat plate with an incoming Mach number ${\mathrm{Ma}}_{\infty }=u_{\infty }/\sqrt{T_{\infty }}=0.1$, temperature $T_{\infty }=1/3$ and Reynolds number Re=ρ∞u∞L/μ=4000, where *L* is the length of flat plate. Since the flow gradients in the transverse *y*-direction are much larger compared to the gradients in the streamwise *x*-direction, the mesh can be stretched in *x*-direction without significantly affecting the accuracy of the results. The computational domain was set to $\left[L_{x}\times L_{y}\right]=\left[200\times 200\right]$ and a rectangular lattice with $\lambda_{x}=2$ was used. The flat plate starts at a distance of $L_{x}/4$ from the inlet and symmetry boundary conditions were imposed at $0\leq x\leq L_{x}/4$. In Figure 9, the horizontal velocity profile at the end of the plate is compared with the results of a regular lattice and with the Blasius similarity solution, where η is the dimensionless coordinate [38],

(74) $$\begin{array}{c} \eta =y\sqrt{\frac{u_{\infty }}{\nu x}}. \end{array}$$

**Figure 10:**
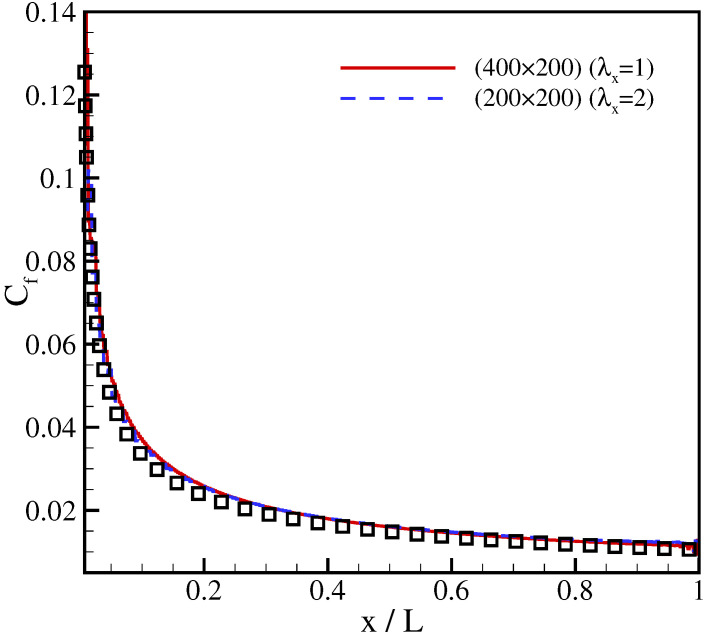
Comparison of the skin friction coefficient for flow over a flat plate at different stretching ratio. Lines: present model; symbols: analytical solution.

It can be seen that results for the regular and the rectangular lattice nearly coincide and agree well with the Blasius solution. Thus, the model achieves accurate results with half of grid points compared to the regular lattice. Furthermore, the distribution of skin friction coefficient over the plate,

(75) $$\begin{array}{c} C_{f}=\frac{\tau_{\mathrm{wall}}}{\frac{1}{2}\rho_{\infty }u_{\infty }^{2}}, \end{array}$$

with the wall shear stress $\tau_{\mathrm{wall}}=\mu {\left(\frac{\partial u}{\partial y}\right)}_{y=0}$, is shown in Figure 10 in comparison with the analytical solution Cf=0.664/Rex, where Rex=u∞x/ν [38]. Also here, the results of the model with the regular and the stretched velocities are almost identical and in good agreement with the analytical solution.

### 3.5. Turbulent Channel Flow

In the final test case, we assess the accuracy and performance of the extended LBM for the turbulent flow in a rectangular channel, for which many numerical [39,40,41] and experimental [42,43] results are available. The channel geometry was chosen as [5.6H×2H×2H], where *H* is the channel half-width. The friction Reynolds number,

(76) Reτ=uτHν,

based on the friction velocity $u_{\tau }=\sqrt{\tau_{w}/\rho }$, was set to Reτ=180. The initial friction velocity was estimated by

(77) $$\begin{array}{c} u_{\tau }=\frac{u_{0}}{\frac{1}{K}\ln Re_{\tau }+5.5}, \end{array}$$

where K=0.41 is the von Kármán constant and $u_{0}=0.1$ is the mean center-line velocity. Periodic boundary conditions were imposed in the streamwise *x*-direction and the spanwise *z*-direction. The flow was driven by a constant body force in the *x*-direction,

(78) g=Reτ2ν2/H3.

In order to accelerate the transition to turbulence, a non-uniform divergence-free forcing field as proposed in [44] was added to the flow for some period of time, until $t^{*}=tH/u_{\tau }=5$.

Similar to the previous test case, grid stretching in *x*-direction with $\lambda_{x}=1.4$ was used in order to reduce the number of grid points in that direction while the temperature was set to T=0.55, same as in Section 3.2

**Figure 11:**
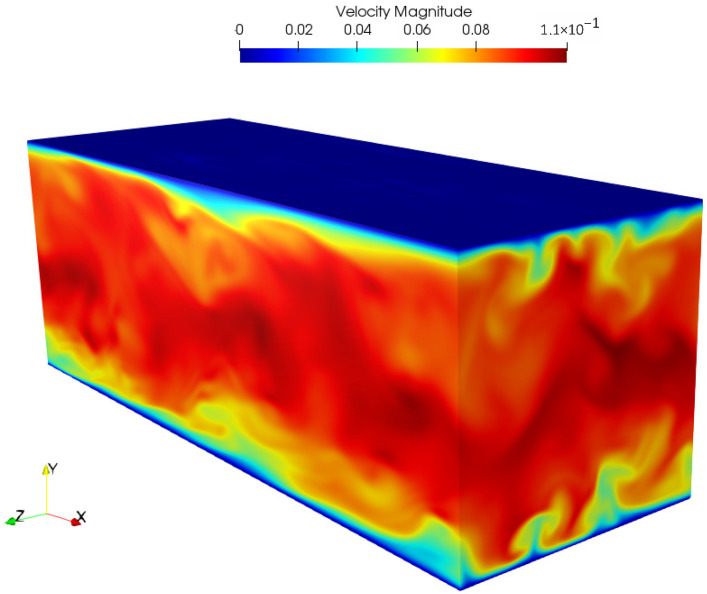
Snapshot of the velocity magnitude in lattice units for turbulent channel flow at Reτ=180 with $\lambda_{x}=1.4$.

A snapshot of the velocity magnitude $\sqrt{u\cdot u}$ is shown in Figure 11.

**Figure 12:**
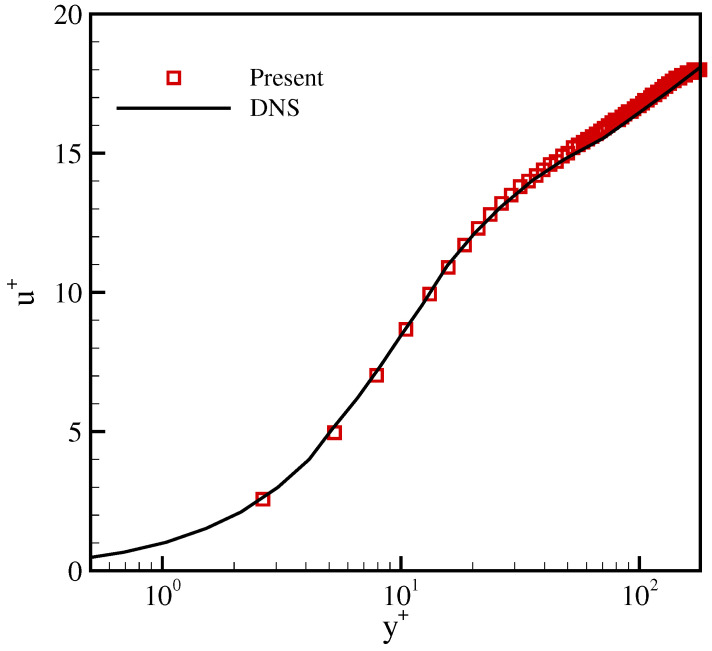
Comparison of the mean velocity profile in a turbulent channel flow at $Re_{\tau }=180$ with $\lambda_{x}=1.4$.

Quantitatively, we compare the mean velocity profile with the DNS results of [40] in Figure 12. In wall units, the mean velocity is given by $u^{+}=\bar{u}/u_{\tau }$ and the spatial coordinate is $y^{+}=yu_{\tau }/\nu$. The statistics are collected after 30 eddy turnover times, i.e., after $t^{*}=30$. It is apparent that the viscous sublayer ($y^{+}<5$), the buffer layer ($5<y^{+}<30$) and the log-law region ($y^{+}>30$) are captured well with our model and the mean velocity profile agrees well with that of the DNS.

**Figure 13:**
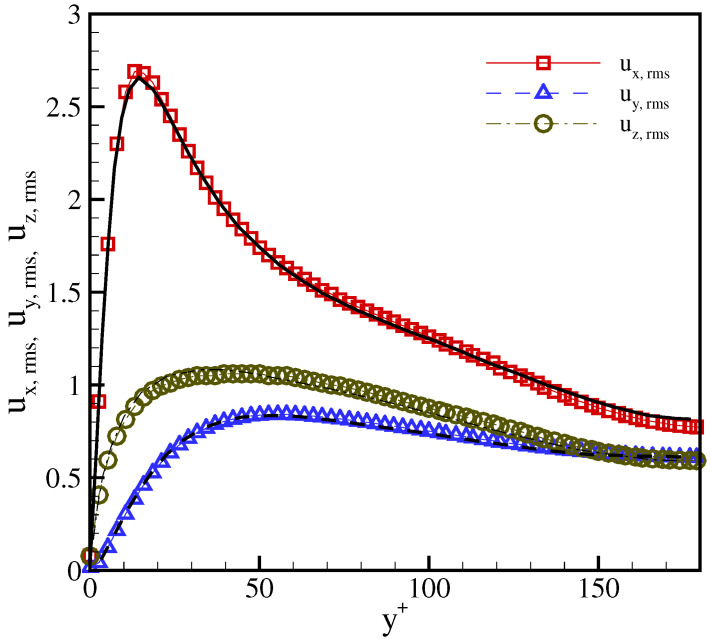
Comparison of the rms of the velocity fluctuations in a turbulent channel flow at $Re_{\tau }=180$ with $\lambda_{x}=1.4$. Symbols: present model; lines: DNS [40].

For a more thorough analysis, we compare the root mean square of the velocity fluctuations with the DNS data in Figure 13. Here, $u_{x,\mathrm{rms}}=\sqrt{\;\bar{\;u_{x}^{{}^{\prime }}u_{x}^{{}^{\prime }}\;}\;}$ and $u_{y,\mathrm{rms}}$ and $u_{z,\mathrm{rms}}$ are defined in a similar way. It can be seen that the results are in excellent agreement with the DNS results [40]. This demonstrates that the LBGK model, also in the presence of a severe anisotropy triggered by stretched velocities, can be used for the simulation of high Reynolds number wall-bounded flows once the corrections are incorporated with the extended equilibrium.

## 4. Conclusions

While even with the standard discrete speeds (5) it is possible to develop an error-free, fully Galilean invariant kinetic model in the co-moving reference frame, it does require off-lattice particles’ velocities [45,46]. Sticking with the fixed, lattice-conform velocities (6), one is faced with an inevitable and persistent error, which spoils the hydrodynamic equations whenever the flow velocity is increased or the temperature deviates from the lattice reference value, or the discrete speeds are stretched differently in different directions. We proposed an upgrade of the LBGK model to enlarge its operation domain in terms of velocity, temperature and grid stretching by suggesting an extended equilibrium. The extended equilibrium is realized through a product-form, which allows us to compensate the diagonal third-order moment anomaly in the hydrodynamic limit by adding consistent correction terms to the diagonal elements of the second-order moment. As a result, the extended LBGK model restores Galilean invariance and temperature independence in a sufficiently wide range, and can also be used with rectangular lattices. Similar to previous proposals [4,5,6,10,12], the relaxation term of the present model remains almost local as it uses only nearest-neighbor information for computation of the first-order derivatives in the extended equilibrium populations. The extended LBGK model was validated in a range of benchmark problems, probing different aspects of anomaly triggered either by increased velocity or temperature deviation from the lattice reference temperature, or by grid stretching. In all cases, the extended LBGK model featured excellent performance and accuracy in both two and three dimensions. In particular, the simulation of homogeneous isotropic turbulence demonstrated the expected speed-up when a higher temperature was used, while simulations of the laminar boundary layer and of the turbulent channel flow using stretched grids demonstrated good accuracy with a reduced number of grid points.

Furthermore, the present model can be extended to other applications including but not limited to high-speed compressible flows, which can be achieved by incorporating another solver for the total energy (see, e.g., the models proposed in [10,12]). Advanced collision models, such as multiple relaxation times (MRT) schemes, can also readily be employed in the present approach, which can be beneficial when running under-resolved simulations. These two avenues shall be subject of further development and application of the extended LBM.

- Note added in proof: After the paper was submitted to peer-review, a later preprint “Central Moment MRT Lattice Boltzmann Method on a Rectangular Lattice” by E. Yahia and K. N. Premnath, arXiv:2103.02119 [physics.flu-dyn], became known to us which adresses a correction for the two-dimensional rectangular lattice in a multiple relaxation time setting.

## Figures and Tables

**Figure A1:**
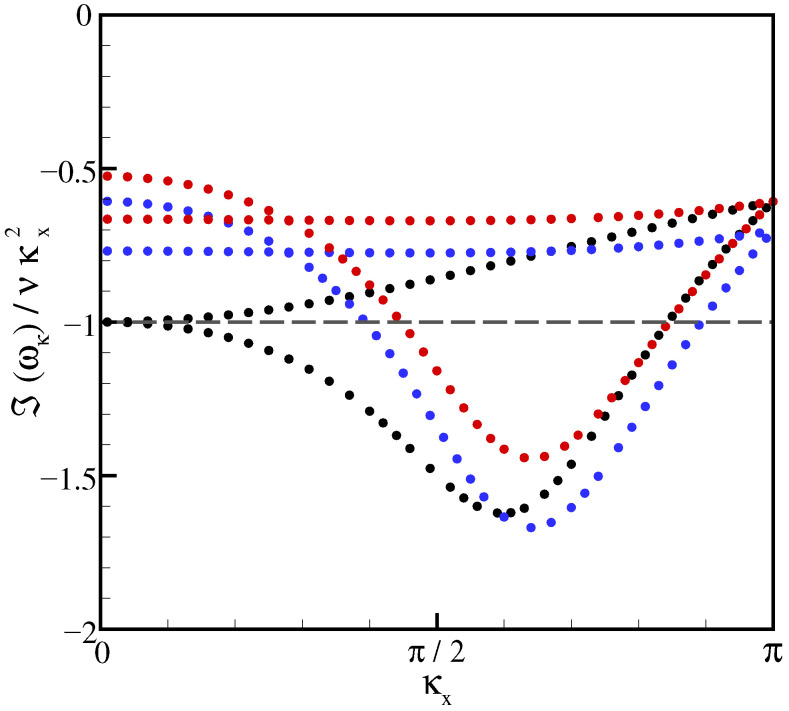
Spectral dissipation of acoustic modes for different models. Red symbols: LBGK; black symbols: extended LBM (33); blue symbols: LC LBM [9]; dashed line: Navier–Stokes. The velocity and temperature are set to $u_{x}=0.3$ and $T=T_{L}$.

**Figure A2:**
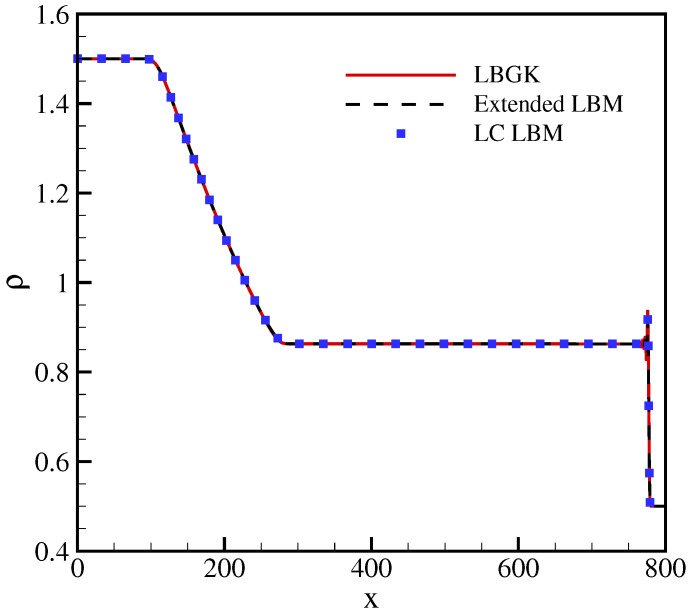
Comparison of density profile for shock tube problem at density ratio $\rho_{l}/\rho_{r}=3$, after 500 iterations. Solid line: LBGK; dashed line: extended LBM (33); symbols: LC LBM [9].

**Figure A3:**
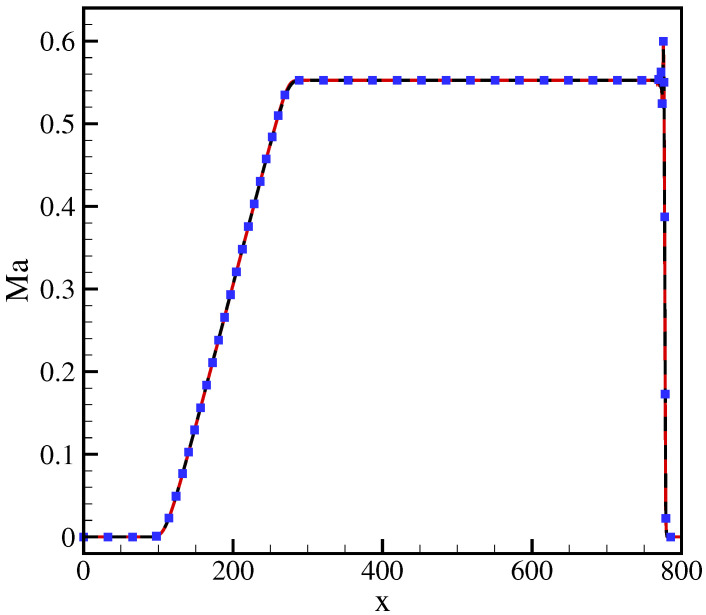
Mach number profile, $\mathrm{Ma}=u/\sqrt{T_{L}}$, for the shock tube problem at density ratio $\rho_{l}/\rho_{r}=3$, after 500 iterations. Solid line: LBGK; dashed line: extended LBM (33); symbols: LC LBM [9].

## Data Availability

Not applicable.

## Abbreviations

The following abbreviations are used in this manuscript:

LBM	Lattice Boltzmann method
LBGK	Lattice Bhatnagar–Gross–Krook

## Appendix A. Comparison of Extended LBGK to Locally Corrected LBM

Below, we compare the locally corrected lattice Boltzmann model (LC LBM) [9] with both the standard and the present extended LBGK. To that end, it suffices to consider the one-dimensional D1Q3 lattice. In order to introduce the LC LBM, we begin with the standard LBGK (δt=1, R=1),

(A1) $$\begin{array}{c} f_{i}\left(x+v_{i},t+1\right)-f_{i}\left(x,t\right)=\omega \left(f_{i}^{\mathrm{eq}}-f_{i}\right). \end{array}$$

The equilibrium populations in (A1) are given by (24) and (25),

(A2) $$\begin{array}{c} f_{i}^{\mathrm{eq}}=\rho \Psi_{i}\left(u_{x},P_{xx}^{\mathrm{eq}},1\right),\;P_{xx}^{\mathrm{eq}}=T+u_{x}^{2},\;i\in \left\{-1,0,1\right\}. \end{array}$$

Thanks to the diagonal anomaly, the second-order asymptotic analysis of Section 2.4 results in the following viscous stress in the one-dimensional version of the Navier–Stokes Equation (61),

(A3) $$\begin{array}{cc} \; & \Pi_{xx}=-2\left(\frac{1}{\omega }-\frac{1}{2}\right)\rho T\partial_{x}u_{x}+{\tilde{\Pi }}_{xx}. \end{array}$$

For the LBGK model, the anomalous (second) term in (A3) reads,

(A4) $$\begin{array}{c} {\tilde{\Pi }}_{xx}^{\mathrm{LBGK}}=-\left(\frac{1}{\omega }-\frac{1}{2}\right)\left[\left(\frac{1-3T}{2T}-\frac{3u_{x}^{2}}{2T}\right)2\rho T\partial_{x}u_{x}+\left(u_{x}\left(1-3T\right)-u_{x}^{3}\right)\partial_{x}\rho \right]. \end{array}$$

Upon realizing that the first term of the anomalous contribution (A4) is similar in its structure to the relevant (first) term in the LBGK stress (A3), the locally corrected (LC) LBGK groups these two terms together and replaces the relaxation parameter ω in (A1) with a new relaxation $\omega_{\mathrm{LC}}$, which depends on the flow velocity. While the original work [9] addressed the case of the lattice temperature, $T=T_{L}=1/3$ (31), we first consider a slightly more general formulation for a flexible temperature parameter. Consequently, the locally corrected relaxation parameter $\omega_{\mathrm{LC}}$ in the LBGK Equation (A1) is defined as,

(A5) $$\begin{array}{c} \frac{1}{\omega_{\mathrm{LC}}}-\frac{1}{2}=\left(\frac{1}{\omega }-\frac{1}{2}\right)X, \end{array}$$

where the renormalization factor *X* reads,

(A6) $$\begin{array}{c} X={\left(1+\frac{1-3T}{2T}-\frac{3u_{x}^{2}}{2T}\right)}^{-1}. \end{array}$$

The LBGK model with the locally corrected relaxation parameter $\omega_{\mathrm{LC}}$ (A5) results in the viscous stress of the form (A3), with the remaining error term,

(A7) $$\begin{array}{c} {\tilde{\Pi }}_{xx}^{\mathrm{LC}}=-\left(\frac{1}{\omega }-\frac{1}{2}\right)X\left(u_{x}\left(1-3T\right)-u_{x}^{3}\right)\partial_{x}\rho . \end{array}$$

For the sake of a discussion, let us introduce the local Mach number, ${\mathrm{Ma}}_{x}=u_{x}/\sqrt{T}$. For a quasi-incompressible (slow) flow, the density variation scales as $\partial_{x}\rho ∼{\mathrm{Ma}}_{x}^{2}$. Thus, for $T\neq T_{L}$, the error (A7) can be estimated as, ${\tilde{\Pi }}_{xx}^{\mathrm{LC}}∼{\mathrm{Ma}}_{x}^{3}$. This is two orders of magnitude lower than the error of the original LBGK at $T\neq T_{L}$, cf. Equation (A4), at small Mach number. Moreover, by setting the temperature $T=T_{L}=1/3$, it was first realized in Ref. [9] that the error (A7) reduces to,

(A8) $$\begin{array}{c} {\tilde{\Pi }}_{xx}^{\mathrm{LC}}=\left(\frac{1}{\omega }-\frac{1}{2}\right)\left(\frac{T_{L}^{3/2}{\mathrm{Ma}}_{x}^{3}}{1-\left(3/2\right){\mathrm{Ma}}_{x}^{2}}\right)\partial_{x}\rho . \end{array}$$

In this case, the scaling at ${\mathrm{Ma}}_{x}\to 0$ becomes, ${\tilde{\Pi }}_{xx}^{\mathrm{LC}}∼{\mathrm{Ma}}_{x}^{5}$. In other words, the local correction at $T=T_{L}$ provides a gain of two orders of magnitude in accuracy with respect to the standard LBGK under the quasi-incompressible flow conditions [9]. This consideration extends straightforwardly to the D2Q9 and D3Q27 lattices by constructing a multiple relaxation time LBM that corrects the relaxation of each diagonal component of the pressure tensor [9].

However, for a generic isothermal flow, the error (A7) becomes amplified through the renormalization factor (A6) as the velocity increases and eventually diverges when $u_{x}^{2}\to (1-T)/3$. This error persists also for the special case $T=T_{L}$ (A8). On the other hand, the second-order analysis of Section 2.4 reveals that the present LBGK with the extended equilibrium (33) removes the entire anomalous term, ${\tilde{\Pi }}_{xx}^{\mathrm{ex}}=0$. Thus, the difference between the extended LBGK and the LC LBM [9] is expected beyond the asymptotic ${\mathrm{Ma}}_{x}\to 0$.

In order to demonstrate this point, a spectral analysis was performed for the two-dimensional D2Q9 lattice (see [21] for details of the spectral analysis in the LBM context). The normalized spectral dissipation of acoustic modes $ℑ\left(\omega_{\kappa }\right)/\nu \kappa_{x}^{2}$, is shown in Figure A1, for $T=T_{L}$ and the background flow velocity $\left(u_{x},u_{y}\right)=\left(0.3,0\right)$, for the three models, the standard LBGK, the present extended LBGK and the LC LBM of Ref. [9].

It can be seen that, the extended LBGK recovers the correct dissipation rate in the continuum limit (vanishing wave number $\kappa_{x}$), confirming its Galilean invariance. However, both the standard LBGK and the LC LBM show deviations in the form of under-dissipation at low wave numbers, while the deviation for the LC LBM is indeed smaller. This non-vanishing deviation is amplified in cases with different working temperature and/or non-unit stretching factor for both the standard LBGK and the LC LBM, which makes their applications limited to the quasi-incompressible flow regime at the lattice temperature.

Finally, it is interesting to note that, in the case of shock capturing, all the three models are expected to behave similarly, given that their respective dissipation rates at the wave number $\kappa_{x}=\pi$ are close in value, see Figure A1. This observation is confirmed by the simulation of a shock tube with the following initial condition,

(A9) $$\begin{array}{cc} \left(\rho ,u_{x},T\right) & =\left\{\begin{array}{cc} (\rho_{l},0,1/3), & x\leq L/2,\\ (\rho_{r},0,1/3), & x>L/2, \end{array}\right. \end{array}$$

with L=800 grid points and viscosity ν=0.04. Results are presented in Figure A2 and Figure A3 for $\rho_{l}=1.5$, $\rho_{r}=0.5$, corresponding to the initial density ratio $\rho_{l}/\rho_{r}=3$. Figure A2 and Figure A3 demonstrate that all models produce almost indistinguishable results, with a similar oscillation pattern at the shock front.
